# A review of the value of quadrivalent influenza vaccines and their potential contribution to influenza control

**DOI:** 10.1080/21645515.2017.1313375

**Published:** 2017-05-22

**Authors:** Riju Ray, Gaël Dos Santos, Philip O. Buck, Carine Claeys, Gonçalo Matias, Bruce L. Innis, Rafik Bekkat-Berkani

**Affiliations:** a GSK, Wavre, Belgium; b Business & Decision Life Sciences, Brussels, Belgium (on behalf of GSK); c GSK, Philadelphia, PA, USA

**Keywords:** influenza, influenza B, mismatch, quadrivalent influenza vaccine

## Abstract

The contribution of influenza B to the seasonal influenza burden varies from year-to-year. Although 2 antigenically distinct influenza B virus lineages have co-circulated since 2001, trivalent influenza vaccines (TIVs) contain antigens from only one influenza B virus. B-mismatch or co-circulation of both B lineages results in increased morbidity and mortality attributable to the B lineage absent from the vaccine. Quadrivalent vaccines (QIVs) contain both influenza B lineages. We reviewed currently licensed QIVs and their value by focusing on the preventable disease burden. Modeling studies support that QIVs are expected to prevent more influenza cases, hospitalisations and deaths than TIVs, although estimates of the case numbers prevented vary according to local specificities. The value of QIVs is demonstrated by their capacity to broaden the immune response and reduce the likelihood of a B-mismatched season. Some health authorities have preferentially recommended QIVs over TIVs in their influenza prevention programmes.

## Introduction

Influenza viruses are enveloped negative-strand RNA viruses that are divided into 3 genera: type A, B and C. The vast majority of human disease is caused by types A and B, which are genetically and structurally similar, but differ in biology, evolutionary and epidemiological framework.^1-3^ Influenza A viruses are subtyped according to 2 surface glycoproteins: haemagglutinin (H) and neuraminidase (N) whereas influenza B viruses form a homogenous group segregated according to 2 antigenically distinguishable lineages (B/Victoria and B/Yamagata).

**Figure 1.**
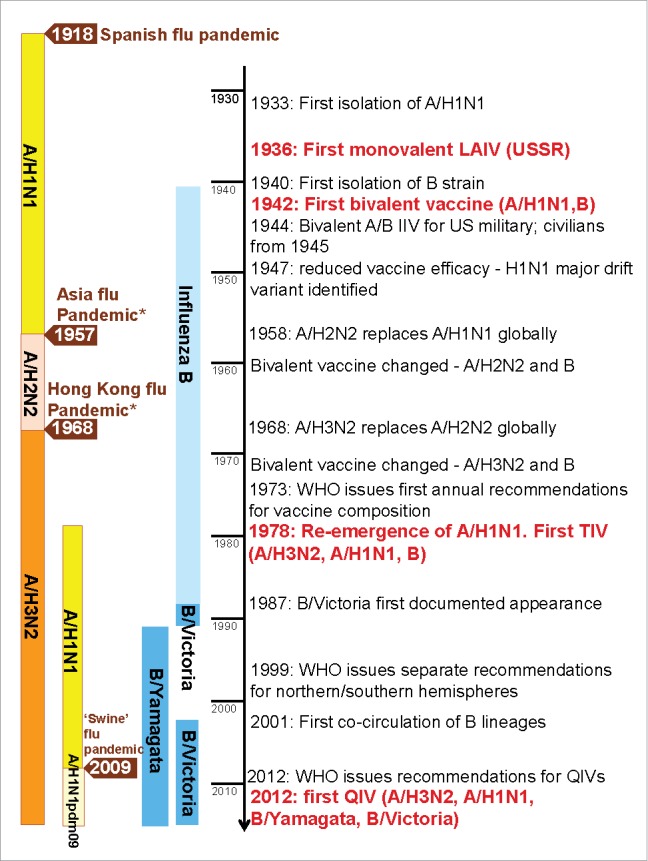
The evaluation of influenza viruses and vaccine development. Information from Hannoun et al.^8^ and McCullers et al.^97^ *Asian influenza pandemic caused by the shifted A/H2N2 strain and Hong Kong influenza pandemic caused by the shifted A/H3N2 strain: both examples of A-strain shift through reassortment (sharing) of genetic material. LAIV = live-attenuated trivalent influenza vaccine; TIV = trivalent influenza vaccine; QIV = quadrivalent influenza vaccine; WHO = World Health Organization.

Influenza viruses undergo constant mutation which enables evasion of existing host immunity leading to recurrent infections that manifest as annual outbreaks.^4^ Periodically, new A subtypes emerge, resulting in a pandemic; the emergent subtype may replace or less frequently co-circulate with the earlier A subtype (Fig. 1). Currently, 2 influenza A subtypes, A/H1N1 and A/H3N2, and the 2 influenza B lineages circulate globally each year.^5,6^ In any influenza season, several influenza types, A-subtypes, or B-lineages may co-circulate, such that the annual influenza burden differs unpredictably from year-to-year, potentially fluctuating from age-group to age-group, and from region-to-region.^4^

Influenza affects all age-groups and while most deaths occur in older adults, influenza deaths also occur in children. In the United States (US), there were 830 reported laboratory-confirmed influenza deaths between 2004–2012 in children < 18 y of age.^7^ Of these, 25% were children aged < 24 months.

Since the development of the first monovalent A/H1N1 influenza vaccines more than 75 y ago, influenza vaccines have always required adaptation in response to changes to provide protection against the predominant circulating influenza viruses (Fig. 1). After influenza B was first isolated in 1940, bivalent (A/H1N1, B) vaccines were developed. The vaccine influenza A subtype was changed in 1958 and in 1969 in response to influenza A shifts that triggered severe pandemics (Fig. 1). Seasonal trivalent influenza vaccines (TIVs) containing 2 influenza A -subtype viruses and one B virus were first produced in 1978 after the re-appearance and co-circulation of A/H1N1 with A/H3N2 viruses (Fig. 1).^8^ Two antigenically distinct lineages of influenza B viruses have circulated globally since 1985 and have co-circulated since 2001.^9^ The influenza B-lineage vaccine strains induce little or no cross-reactive protection against the alternate B-lineage,^10,11^ such that for TIVs, protection against the circulating influenza B lineage relies on correctly predicting the B-lineage likely to predominate in the upcoming season. The degree of similarity or difference between the circulating viruses and the strains included in the vaccines is often referred to as “vaccine match” or “vaccine mismatch.” A review of influenza B in 26 countries concluded that the type B lineage selected for inclusion in the annual vaccine differs from the predominant circulating lineage in around 25% of seasons.^12^ In a mismatched season, influenza vaccine effectiveness may be suboptimal against influenza B epidemics, potentially leading to an increased public health burden during those seasons.^13-18^ This observation led to international cooperation among the scientific community, leading to the development of quadrivalent seasonal influenza vaccines (QIVs) that included both of the circulating influenza B lineages.^10,19^ In this work we reviewed the development of currently licensed QIVs and provide an overview of their societal value by focusing on the preventable disease burden associated with vaccination. We identified modeling studies estimating the potential impact of QIVs compared with TIVs in terms of clinical outcomes prevented.

## Clinical evaluation and characteristics of currently licensed of QIVs

The first QIV was licensed in 2012 and currently 3 manufacturers produce QIV in various forms (inactivated [IIV4] or live-attenuated [LAIV4]), with new vaccines under development.^20,21^ Seasonal influenza vaccines induce antibody responses against the head of the haemagglutinin glycoprotein. An haemagglutinin inhibition (HI) titer of ≥ 1:40 is generally accepted as indicative of clinical benefit since it has been associated with protection from influenza illness in up to 50% of subjects.^22,23^

Cumulative evidence for the immunogenicity and safety of QIVs was obtained through studies designed to demonstrate non-inferiority in terms of the HI geometric mean antibody titres (GMTs) and seroconversion rates (SCRs) to the common 3 strains in the candidate QIVs compared with licensed TIVs, and to demonstrate superiority in terms of the HI GMT and SCR of the added influenza type B lineage compared with licensed TIVs containing either the B/Yamagata or B/Victoria lineages. Safety and reactogenicity of QIVs versus TIVs were also assessed.

### IIV4s manufactured by GSK

GSK produces IIV4s that are identical in antigen content but manufactured and licensed separately: *FluLaval Quadrivalent* (Q-IIV4) is manufactured in Quebec, Canada, and *Fluarix Quadrivalent* (D-IIV4) is manufactured in Dresden, Germany.^24^ Both vaccines are approved for use in individuals from 3 y of age. Q-IIV4 is also licensed from 6 months of age in Canada, the US, and Mexico. Individuals from the age of 6 months through to adults > 65 y were enrolled in the clinical development program.^24^ The results of key studies have been recently reviewed.^24-26^

*Fluarix Quadrivalent* (*Fluarix Tetra, Influsplit Tetra* and *Alpharix Tetra*): In studies in adults, adolescents and children (≥ 3 y of age), HI GMTs and SCRs following D-IIV4 were non-inferior to licensed D-IIV3s for common strains, and superior in terms of the HI GMT and SCR for the additional type B lineage. Addition of the fourth strain had no impact on the reactogenicity and safety profile of the vaccine.^27,28^ Efficacy of D-IIV4 is supported by the demonstrated efficacy of trivalent *Fluarix* in healthy adults aged 18–64 y; since both vaccines use the same manufacturing processes. In adults, *Fluarix* demonstrated statistically significant efficacy of 66.9% against culture-confirmed antigenically-matched influenza A and/or B (95% confidence interval [CI] 51.9–77.4).^29^

*FluLaval Quadrivalent (Flulaval Tetra)*: In studies in adults, adolescents and children (≥ 3 y of age), the immunogenicity of Q-IIV4 was non-inferior to that of licensed IIV3s for common strains, and superior for the additional type B strain from the alternate lineage in terms of HI GMTs and SCRs. Similar to D-IIV4, addition of the fourth strain did not impact the reactogenicity and the safety profile of Q-IIV4 as compared with IIV3s.^30,31^

In children 6 to 35 months of age, Q-IIV4 (containing 15 μg of each virus strain) was non-inferior to *Fluzone Quadrivalent* (F-IIV4, Sanofi Pasteur) (containing 7.5 μg of each virus strain) for each vaccine strain, and superior in terms of the immune response to both influenza B strains in 6–17 month old children and unprimed children of any age.^32^

Efficacy of Q-IIV4 was demonstrated in children aged 3−8 y (N = 5,220) who were randomized to receive either Q-IIV4 or inactivated hepatitis A vaccine as control.^33^ Efficacy of Q-IIV4 in preventing influenza of any severity was 55.4% (95% CI 39.1–67.3), and efficacy against moderate-to-severe influenza (defined as a body temperature > 39°C, acute otitis media, lower respiratory tract illness, or serious extra-pulmonary complications) was 73.1% (97.5% CI 47.1–86.3).^33^ Influenza cases in the Q-IIV4-vaccinated group tended to be of mild clinical severity and were associated with substantially lower numbers of medical visits, hospitalisations, school absences and parental absences from work than episodes of influenza in the control group.^33^

### IIV4s manufactured by Sanofi Pasteur

Sanofi Pasteur manufacturers 2 IIV4s administered either intramuscularly (F-IIV4 licensed for use from 6 months of age) or intradermally (*Fluzone Intradermal* Q*uadrivalent* [intradermal F-IIV4] licensed for individuals between 18–64 y of age).

*Fluzone Quadrivalent*: F-IIV4 was evaluated in 3 clinical trials enrolling > 5,500 participants aged 6 months-9 y, ≥ 18 y and ≥ 65 y, respectively.^34-36^ Non-inferiority was demonstrated between F-IIV4 and IIV3s in terms of HI GMTs and SCRs for common strains in all of these age-groups; except for the SCR for the H1N1 strain in subjects aged ≥ 65 y. The failure to meet this non-inferiority criterion may be related to a high baseline prevalence of seropositivity against this strain.^34^ Superiority of the immune response to the added influenza type B lineage compared with 2 licensed IIV3 formulations in terms of GMTs and SCRs was also demonstrated in all age-groups, except for the GMT ratio for the B/Victoria lineage strain in adults aged ≥ 65 y. At least 73.2–100% of adults and 66.9%–98.8% of children who received F-IIV4 had HI titres ≥ 40 after vaccination. The safety profile of F-IIV4 was similar to that of the studied IIV3s.^34^

*Fluzone Intradermal Quadrivalent*: This intradermal vaccine is licensed for use in adults aged 18–64 y and was evaluated in 3360 participants in one study in the US.^37^ Non-inferiority was demonstrated between the intradermal F-IIV4 and intradermal IIV3 in terms of HI GMTs and SCRs to common strains, and superiority of the immune response to the added influenza type B lineage compared with 2 intradermal IIV3 formulations was also demonstrated. At least 86% of intradermal F-IIV4 recipients had HI titres ≥ 40 after vaccination.^37^ The safety profile of intradermal F-IIV4 was similar to that of intradermal IIV3s.

### LAIV4s manufactured by AstraZeneca

*Flumist Quadrivalent* (US, Canada) and *Fluenz Tetra* (European Union) are licensed for use in individuals from 2–59 y internationally and 2–49 y in the US. Two clinical trials conducted in the US evaluated immunogenicity and safety in subjects aged 2–49 y.^38-41^ Non-inferiority of the HI antibody response to LAIV3s was demonstrated for common strains. Post-hoc analyses demonstrated superiority of the HI antibody response for the added B lineage.^38^ The safety and reactogenicity of LAIV4s were similar to LAIV3s.

## The value of QIVs in reducing the burden of influenza B – review of the literature

Influenza B causes epidemics approximately every 2–4 years^42,43^ that impact all age-groups but proportionally more older children.^43-47^ Influenza B has been reported to be clinically indistinguishable to influenza A,^48-50^ and has been linked to severe disease, including encephalitis, myositis, pneumonia and fulminant disease in children.^51-54^ The majority of subjects with lethal influenza B in a US case series died before they could be hospitalised, highlighting the importance of vaccine prophylaxis in mitigating this risk.^54^

A systematic review of the literature describing influenza B disease published between 1995 and 2010 concluded that influenza B was more likely to be severe in children than in adults.^55^ Although influenza B affects all age segments, it is more common among children aged 5–17 y.^12,55^ Influenza B accounts on average, for approximately 20–30% of influenza isolates from respiratory samples across seasons,^12,56^ although the reported frequencies vary from year-to-year and from region-to-region, ranging from 0–62.9% in children and 0–48% in adults.^55^ The Danish 2015/16 influenza season was characterized by a major (88%) B-lineage mismatch and it has been put forward that morbidity during the season may have been lower if QIVs had been used instead of TIVs.^18^ More recently, a comprehensive review of the influenza B burden in 9 European countries highlighted the scarce attention that the influenza B burden has received over previous decade as compared with influenza A.^57^ This works also underscores the lack of predictable patterns in strain circulation seen in these countries and thus the continuous risk of mismatch when there is high influenza B circulation.

For QIVs, modeling studies are sometimes used to assess cost-effectiveness and whether the added value of the vaccine is likely to offset the added cost; models may also contribute information about the societal value of QIVs quantifying the number of preventable influenza outcomes compared with TIVs. The reported added value of QIVs comes from its capacity to provide broader immunity against influenza B, thereby reducing the likelihood of a mismatched season.

**Figure 2.**
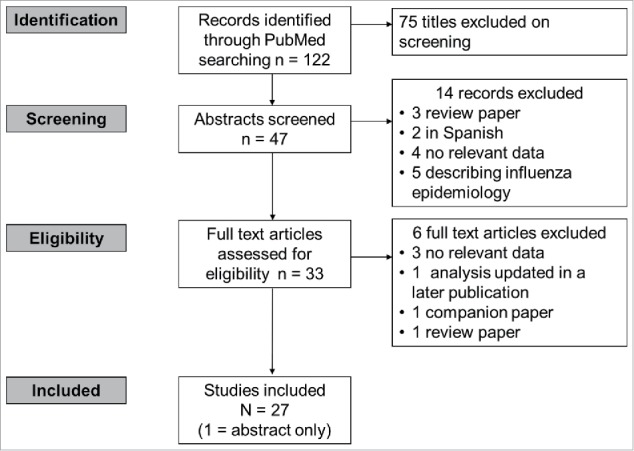
Results of the literature search (30 October 2016). Search string: “(Quadrivalent OR tetravalent) (influenza vaccine OR flu vaccine) (cost OR burden OR epidemiology OR death OR mortality OR illness OR hospitalisation OR hospitalization)” No limits applied.

We systematically queried the PubMed database for papers reporting the potential value of QIVs compared with TIVs in terms of illness, hospitalisations and deaths averted using the search string provided in Fig. 2. Articles were selected by a 3-step selection procedure based on 1) screening of title and abstract, 2) screening of full-text article, and 3) final screening during the data-extraction phase. The titles and abstracts retrieved from the Pubmed database were screened in duplicate by 2 independent researchers. The results were compared and discussed; all selected references from the 2 researchers were included for full text selection.

In case of discrepancy or disagreements during the selection, a third researcher was consulted and the study was discussed until consensus was reached. If articles reported on the same study, the most relevant or most complete article was included in this review. If articles complemented each other, both articles were included. The reasons for exclusion of full-text papers were recorded.

We identified 27 eligible studies from 14 countries that used multiple modeling methods and highly variable background assumptions to estimate the potential impact of QIVs over TIVs in various immunisation scenarios (Table 1). All of the estimates were highly dependent upon the choice of baseline data, such as the assumed degree of cross-protection afforded by TIVs and the level of mismatch in a given season. In all cases, the results of more conservative static models (i.e., that could not adjust for potential herd protection or reduction in disease transmission) were lower than dynamic models that included herd-protection or disease transmission effects in the model. A summary of current disease burden information and the potential impact of QIVs on the influenza burden from modeling studies is presented for selected countries below.

**Table 1.** Summary of QIV modeling data in different countries: results from a review of the literature.

	Model	Vaccinated		Time	Influenza vaccine	Illness averted by QIVs compared with TIVs		
	framework	population	Population size	horizon	coverage (%)	Total Cases	Total hospitalisations	Total Deaths
**North America**								
US ^58^	Static	All ages	270–304 million	10 y	18–30^*^	2,741,575 (Range 2200–970,000)	21,440 (Range 14–8,200)	1371 (Range 1–485)
US ^59^	Static	All ages	311.6 million	1 y	21.3–66.6^**^	30,251	3,512	722
US ^60^	Static	≥ 65 y	41.5 million	1 y	67	26,701	1,345	211
US ^61^	Static	≥ 65 y	44,704,074	10 months	64.7	39,136	1,648	458
US ^65^	Dynamic	All ages	US population	10 y	∼25–60^**^	1,973,849 annually	—	1,396 annually
US ^64^	Dynamic	All ages	313.9 million	1 y	46.2	1,382,509	18,354	2,981
US ^62^	Dynamic	All ages	US population without immigration	13 y	Weekly age-based from CDC	6,267,800 (Average 482,000 annually)	—	—
US ^63^	Dynamic	All ages	US population	20 y	Weekly age-based from CDC	16 million	137,645	16,199
Canada ^68^	Dynamic	All ages	34.8 million	10 y	16.1–64.4^**^	135,538 annually	1876 annually	328 annually
Ontario, Canada^67^	Static	All ages	12.8 million	2000–2008	27–81^**^	2,516 annually	27 annually	5 annually
**Europe**								
UK ^68^	Dynamic	All ages	62.8 million	10 y	17.6–71.1^**^	88,755 annually	1050 annually	230 annually
UK ^71^	Static	≥ 65 and risk groups	63.7 million	100 y	34.07–100^**^	1.4 million	41,780	19,906
				10 y		183,844	4,871	2,142
Germany ^73^	Dynamic	0–15 y	Approx. 80 million	20 y	26.8–33.4^*^	79,000 annually	—	—
		16–60 y				223,000 annually	—	—
		≥ 61 y				93,000 annually	—	—
		All ages				395,000 annually	—	—
Germany ^74^	Dynamic	All ages	81.3 million	20 y	Not given	276,505 annually	5,690 annually	262 annually
Finland ^75^	Dynamic	All ages	Finnish population	20 y	∼10–75^**^	40,500 annually†	360 annually†	54 annually†
Belgium ^76^	Dynamic	2–17 y^††^	11.16–11.63 million	10 y	50	2,953,995	16,968	1,455
Spain^77^	Static	≥ 65 y, at risk ≥ 3y	46,727,891	100 y	0–72.47^**^	18,565 in the first year	407 in the first year	181 in the first year
Italy^78^	Static	All ages at risk, ≥ 65y	17,420,318	2014–2015	9% QIV, 31.02 total	2,632	100	—
France^79^	Static	All ages	French population	2003–2012	Not reported	6,214 consultations	614	372
5 countries in Europe^80^	Static	6 m-2 y		2002–2013	6.1–19.2	28,877	140	0
		2–17 y			4.1–14.0	219,163	348	4
		18–49 y high risk			30.9–52.0	83,635	389	79
		18–49 y low risk			6.5–12.0	133,656	110	0
		50–64 y high risk			30.9–52.0	89,908	1,801	325
		50–64 y low risk			14.9–25.3	86,216	515	0
		65+			48.6–69.2	393,270	21,151	9,391
		All ages				1,034,727	24,453	9,799
		All ages	Extrapolated to 27-EU			1,624,533	37,317	14,866
**Asia-Pacific and Western Pacific**								
Hong Kong ^83^	Static	All ages	7.2 million	1 y	11.0–39.1^**^	91/100,000	1.8/100,000	0.046/100,000
Hong Kong ^98,99^	Static	≥ 65 y	747,000–924,000	2001–2010	39.1	191.3/100,000	—	—
		65–79 y	601,000–666,000			104.8/100,000	0–2.4/100,000	0–0.14/100,000
		≥ 80 y	146,000–258,000			451.4/100,000	0–13.1/100,000	0–0.77/100,000
		All ages	Not reported			25.6/100,000	—	—
Albany, Australia ^85^	Static	All ages	ABS CCD	2003–2013	2–20 (selected range)	0.1/100,000 (3.8% reduction)	2.0/100,000 (2.2% reduction)	0.1/100,000 (2.1% reduction)
Australia ^86^	Static	6–59 months	173,778	2002–2012	41.3	4,153	23	0
		5–17 y	509,239		41.3	11,824	11	0
		18–49 y	2,223,249		36.2	10,240	86	1
		50–64 y	1,296,098		36.2	5,731	143	6
		65+ y	3,221,312		74.6	36,322	3,257	675
		Total	7,423,676 at risk		—	68,271	3,522	683
Thailand ^100^	Static	All ages	66–68 million	2007–2012	3–12^*^	21, 974	698	7
Taiwan ^101^	Static	All ages	Not reported	100 y	0–39.49^**^	529,874	8,126	3,590
**Africa**								
Agincourt, South Africa ^85^	Static	All ages	40,383	2003–2013	2–20 (selected range)	0.3/100,000 (12.0% reduction)	4.8/100,000 (18.6% reduction)	2.0/100,000 (17.6% reduction)

* Coverage across seasons

** coverage across age ranges, ^††^this study evaluated immunisation of 2–17 y olds with QIV vs. the baseline scenario of TIV vaccination of at-risk groups, †switching from TIV to Q-LAIV in 2–18 y olds (with QIV in other age groups) was estimated to prevent an additional 76,100 infections, 540 hospitalizations, and 72 deaths compared with TIV

ABS CCD = Australian Bureau of Statistics Census Collection Districts; CDC = Centers for Disease Control and Prevention in the United States; ONS = Office of National Statistics; QIV = quadrivalent influenza vaccine; TIV = trivalent influenza vaccine; US = United States; UK = United Kingdom; y = years

### United States

A prospective study of medically-attended visits for influenza-like-illness (ILI) in the US from 2009–2013 identified influenza B in 29.0% of influenza-positive respiratory specimens from patients of all ages attending outpatient clinics.^56^ In each study year the incidence of visits for ILI caused by influenza B was highest in 5–17 year-olds (range 1.0–13.3/1000 population between 2010–2013).^56^ Retrospective studies reported that around 42% (n = 6,084,951) of annual influenza-attributable office visits in < 65 y olds (2001–2009), and 30% of annual influenza-attributable hospitalisations in all ages (1997–2009), were attributable to influenza B (mortality rate 30/100,000 population).^45,47^ In 4 of 12 seasons encompassed in one study, 51–95% of all influenza-associated deaths were attributed to influenza B.^45^

The impact of using QIVs as compared with TIVs in the US was estimated in 7 studies (Table 1). Reed et al.,^58^ estimated that over the 10-year period between 1999 and 2009, QIVs could have prevented 2.7 million more influenza cases, 21,440 more hospitalisations and 1,371 more deaths than TIVs. The preventable burden each year ranged from 2,200–970,000 illnesses, 14–8,200 hospitalisations and 1–485 deaths. The all-age estimate obtained by another static model appeared similar, suggesting that QIVs would prevent 30,251 more influenza cases annually than TIVs.^59^

Using data from a study that compared the difference between vaccination with QIVs vs. no vaccination and vaccination with TIVs vs. no vaccination, it can be estimated that in one average year, use of QIV rather than TIVs among ≥ 65-year-olds, would prevent 26,701 illnesses, 1,345 hospitalisations and 211 deaths in this age-group.^60^ A second study that considered adults aged 65 y and older estimated that using QIVs instead of TIVs would prevent 39,136 additional influenza cases, 1,648 additional hospitalisations and 458 additional deaths, annually.^61^ Differences in the model and in the underlying assumptions likely account for the different estimates.

Four studies used dynamic models that incorporated effects of disease transmission and herd-protection in the model (Table 1). As a result, the estimates obtained from these models were higher than those from more conservative static models: Crépey et al.,^62^ estimated that use of QIVs in the US would have averted 15.8% more influenza B cases, or more than 6.2 million influenza cases (average 482,000 per year), than TIVs over the 13-year study period (2000–2013). Using a similar model, de Boer et al., estimated that replacing TIVs with QIVs in the next 20 y (2014–2034) would reduce influenza B cases by 27.2%, or 16 million cases.^63^ Mullikin et al.,^64^ calculated influenza illnesses prevented by TIVs, QIVs (or an adjuvanted TIV in elderly with TIV in other ages) compared with no vaccination, and estimated that in an average season with an average vaccine match (obtained using 1999–2014 US data), vaccination with QIVs would prevent 1,382,509 more illnesses than vaccination with TIVs (an estimate which is higher than the upper range of that from Crépey et al.^62^), 18,354 hospitalisations and 2,981 deaths. Brogan et al.,^65^ estimated that over a 10-year period, QIVs instead of TIVs would prevent 1,973,849 additional influenza cases and 1,396 deaths annually. These studies illustrate that although the impact of QIVs may vary in any influenza season, the multi-year cumulative benefit of QIVs over TIVs are predicted to result in substantial societal benefit. This is particularly true for a dynamic model that accounts for herd immunity.

### Canada

B-lineage mismatches between the vaccine and circulating strain occurred in 7 out of 15 seasons in Canada between 2001–2015.^4^ During the 2011/12 influenza season the mismatched influenza B lineage contributed substantially to serious outcomes in adults; the hospital-based Serious Outcomes Surveillance Network in Canada identified influenza B in 65% of adults hospitalised with influenza, of which 68% (205/383) were due to a vaccine mismatched lineage.^66^ Around 12% of influenza B cases required admission to intensive care. The 30-day mortality was 3% for the vaccine-matched lineage and 10% for the mismatched lineage.

Two studies estimated the potential impact of QIVs over TIVs in Canada: one used a static model and the other a dynamic model. The static model estimated that use of QIV would prevent 2,516 cases, 27 hospitalisations and 5 deaths annually in addition to TIVs.^67^ The dynamic model estimated that use of QIVs would prevent 135,538 cases, 1,876 hospitalisations and 328 deaths annually in Canada compared in addition to TIVs.^68^

### United Kingdom (UK)

Modeling studies conducted over 12–14 seasons (1995/1996/1997–2009) with results extrapolated to the total UK population estimated seasonal rates for general practitioner visits, hospitalisations and deaths attributable to respiratory disease caused by influenza B of 355/100,000, 2/100,000, and 1/100,000 population respectively.^43,69^ Around one-third of visits to a general practitioner for otitis media were attributed to influenza type B.^43^ The number and rate of hospitalisations due to influenza B-attributable respiratory disease was highest in 5–17 year-olds and exceeded that due to influenza A in some seasons.^69^

Over a 13-year period in Scotland (2000–2012), influenza B detections were close to, or exceeded influenza A detections for 6 out of 13 seasons.^70^

Two studies in the UK have compared the impact of TIVs and QIVs in those ≥ 65 y of age and clinical at-risk groups, and its implementation in the whole population.^68,71^ In a static model that considered vaccination of elderly and at-risk groups, QIVs are expected to prevent a total of 183,844 cases, 4,871 hospitalisations and 2,142 deaths due to influenza B over a cumulative 10-year time horizon.^71^ A separate study using a dynamic model reported that vaccination of all age-groups (current vaccine coverage rates) was estimated to prevent 88,755 cases, 1,050 hospitalisations and 230 deaths due to influenza B annually.^68^

### Germany

In a prospective surveillance study of children ≤16 y of age hospitalised with acute respiratory infection, influenza B was associated with pneumonia in 36% of cases (5/14) and by myositis in 7% (1/14).^72^ In the same study, influenza A infection was associated with pneumonia in 25% (26/102) of cases, otitis media in 25% (26/102) of cases, anaemia and syncope each in one case.

Two studies used a simulation model that incorporated cross-immunising events and waning/boosting of immunity over a 20-year time horizon.^73,74^ One study estimated that compared with TIVs, QIVs would prevent 11.2% more influenza B cases. ^73^ Each year this would reduce the annual number of influenza cases by 3.6% in 0–15 y olds, 4.0% in 16–60 y olds, 6.9% in those aged ≥ 61 y and 4.3% (or 395,000 cases) overall.^73^

The second study estimated that QIVs would prevent 4% more influenza cases, 5.7% more hospitalisations and 6.4% more deaths than TIVs.^74^

### Finland

A dynamic model using data from the 2000 to 2009 influenza seasons estimated that in the Finnish population, QIVs would prevent 40,500 cases, 360 hospitalisations and 54 deaths each year compared with TIV.^75^ The number of cases was estimated to be even further reduced if the QIV used in 2–18 y olds was LAIV4.

### Belgium

Extending the current Belgian influenza immunisation strategy from individuals at-risk (including those aged 50 y or over) to include children 2–17 y of age was assessed in a dynamic model.^76^ Assuming 50% coverage of QIVs among 2–17 y olds, 2.95 million cases of influenza were calculated to be averted over a 10-year period compared with no vaccination of this age group. Most (63%, or 1,869,582) of these averted cases were due to indirect effects in adults in whom vaccine coverage rates with TIV were assumed to be unchanged. Of a total of 16,968 averted hospitalisations and 1,455 averted deaths, 11,567 and 1,455, respectively, were in adults.

### Spain

Switching from TIVs to QIVs in at-risk individuals from 3 y of age and in those aged 65 y and over was estimated to prevent an additional 18,565 influenza cases, 407 hospitalisations and 181 deaths in the first year after implementation.^77^

### Italy

QIVs were introduced in Italy for the 2015/16 season. Assuming that QIVs were used in 9% of the population targeted by the national immunisation program (all individuals at risk and those aged ≥ 65 y), 1,601 additional cases of uncomplicated influenza and 1,031 additional cases of complicated influenza were estimated to be averted compared with TIVs used alone.^78^

### France

By switching from QIVs to TIVs in France during an average epidemic season between 2003 and 2012, it was estimated that 6,214 consultations for influenza, 614 additional hospitalisations and 372 deaths would have been averted.^79^

### European Union

A static model using data from France, Germany, Italy, Spain and the UK, estimated that replacement of TIVs with QIVs during 2002–2013 (2009 season excluded) would have prevented 1,034,727 additional influenza cases, 24,453 hospitalisations and 9,799 deaths.^80^ Extrapolation to the 27-EU suggested that QIVs would have prevented an estimated 1,624,533 influenza cases, 37,317 hospitalisations and 14,866 deaths. The majority of hospitalisations and almost all deaths prevented would have been in high-risk groups or the elderly.

### Hong Kong

Using hospital records to identify laboratory-confirmed influenza cases between 2000–2010, Chan et al.,^81^ estimated that the average annual incidence of hospital admission (all ages) due to influenza B was 20.6/100,000 population. The highest annual hospitalisations rates were in children < 5 y and 5–9 y of age (median 238/100,000 and 152/100,000, respectively). Both influenza B lineages co-circulated for 9 out of the 10 study years. In the 6 y where a single influenza B lineage predominated (defined as > 80% of identified strains), a mismatch between the predominant B-strain and the vaccine strain occurred in 4. Modeling using Hong Kong death statistics and sentinel laboratory surveillance between 1998–2009 estimated an annual excess mortality of 2.5/100,000 person-years due to influenza B, increasing to 20.3/100,000 person-years in adults aged ≥ 65.^82^

Static models estimated that compared with TIVs, QIVs used at all ages would prevent 91 influenza illnesses per 100,000 population, 1.8/100,000 hospitalisations and 0.046/100,000 deaths over 1 y (Table 1).^83^ Vaccination of ≥ 65 year-olds with QIV between 2001 and 2009 is estimated to have prevented 191.3 influenza illnesses per 100,000 population in elderly, with the highest reduction in those aged ≥ 80 y (reduction of 451.4 illness per 100,000 population).

### Australia

The 2015 influenza season in Australia was dominated by influenza B, which accounted for 62% of all cases notified;^84^ 38% of influenza B isolates were the B/Victoria lineage not included in the 2015 southern hemisphere TIVs. The highest influenza B notification rate was in children 5–9 y of age, followed by 0–4 y and 0–14 y.^84^

A static model using 2003–2013 data from a single town in Western Australia estimated that compared with TIVs, QIVs would reduce influenza illness by 3.8%, hospitalisations by 2.2% and deaths by 2.1%.^85^ The authors noted that there was a good match between the vaccine and circulating lineages during the study period in Australia, under which scenario the benefit of QIVs over TIVs is marginal.^85^ The same study also investigated the impact of QIVs over TIVs in a rural area of South Africa, and reported a greater impact due to a higher degree of mismatch in the seasons studied (Table 1).

A second static model estimated that the use of QIVs instead of TIVs in children and adults at-risk for influenza (eligible for free vaccination as defined in the Australian Immunisation handbook) between 2002 and 2012, would have reduced influenza cases, hospitalisations and deaths by 1.02% (68,271), 2.4% (n = 3,522) and 3.7% (n = 683), respectively.^86^ The highest impact of QIV was in young children and the elderly. In adults aged 65 y and over, QIVs were estimated to prevent an additional 10.1 hospitalisations and 2.1 deaths per 100,000 person-years over TIVs.

## Current recommendations for QIVs

Since licensure of the first QIV in 2012, an increasing number of countries use QIVs either permissively (TIVs or QIVs), or preferentially (TIVs available but QIVs preferred) (Table 2). However, while several supranational organisations acknowledge that QIVs may improve protection against influenza B strains compared with TIVs, at this time, most health authorities do not preferentially recommend one over the other (Table 2). Health authorities are generally concerned that with a trend toward an increased influenza vaccine uptake overall, preferential recommendations may be counterproductive if a preferred product is not sufficiently manufactured and supplied to cover the entire population to be vaccinated.

**Table 2.** Current recommendations for influenza vaccination using quadrivalent seasonal influenza vaccines (QIVs).

Countries/authorities with permissive recommendations for QIVs use	Year recommended	Age/group indicated for QIVs
World Health Organization ^102^	2012	Pregnant women, Children <5 y, Health care workers, Elderly >65 y, Chronic conditions
Germany ^103^	2013	Pregnant women, Children <5 y, Health care workers, Elderly >65 y, Chronic conditions
United States ^104^	2013	Children (≥6 months) & Adults
Hong Kong ^105^	2013	Children (≥ 3 y) & Adults
Canada ^106^	2014	From 6 months of age
Italy ^107^	2014	Children (≥ 3 y) & Adults
France ^108^	2014	Children (≥ 3 y) & Adults
Belgium ^109^	2015	From age 2 y
Brazil ^110^	2014	Elderly 60+ y
Countries preferentially recommending QIVs		
United Kingdom ^111^	2013	Children 2–7 y and children at risk 2–18 y.
Germany ^112^	2014	All long-distance travelers
Brazil ^110^	2015	Elderly
Australia ^113^	2015	From 6 months of age

QIV = quadrivalent seasonal influenza vaccine (LAIV or IIV4)

After successful pilot programmes in 2013 and 2014, influenza vaccination using LAIV4 is now offered to 2–4 y old school children in the UK, as well as to at-risk groups from 6 month to 17 y of age.^87^ This innovative initiative aims to reduce disease incidence in children and transmission to older age-groups, thereby achieving societal benefit at the overall population level.^88^

## Conclusions

Although varying from year-to-year, on average, influenza B causes up to one-third of influenza infections each season. A large body of evidence from numerous countries demonstrates that influenza B accounts for a significant proportion of the overall burden of influenza that inundates healthcare services annually. Once thought to cause predominantly mild illness, numerous studies now indicate that there is little difference in the clinical symptomatology and outcomes of influenza B vs. A. Hospitalisations and mortality attributable to influenza B may have previously been underestimated, with studies reporting higher mortality following influenza B infection than A in some years.^13,89-91^ In parallel, the 2 influenza B lineages frequently co-circulate, and due to the complexity involved in accurately forecasting which B viruses will circulate, mismatches between the B strain selected for TIVs and circulating strains have occurred in up to one-half of seasons.^4,92^ Evidence from clinical trials and observational studies suggest that B-mismatched seasons are accompanied by a higher public health burden than well-matched seasons.^15,51^ Although the risk of breakthrough influenza A from vaccine strain mismatch remains, the risk of breakthrough influenza B from vaccine lineage mismatch can be eliminated by QIV.

Brazil is unique with regards to the pattern of influenza and viral circulating in this heterogeneous climate. A recent review paper highlighted that over a 9 y follow-up period, influenza B lineages circulated in 3 seasons, of which, during one season, there was a high degree of mismatch between the vaccine lineage and the predominant circulating lineage (91.4% [2013]).^93^ Within tropical and sub-tropical regions such as Brazil, influenza B can have protracted circulation patterns and co-circulation of both B-lineages may not be uncommon, thus further highlighting the added value of QIVs in these countries.^94^

Modeling the impact of QIVs over TIVs is challenging, not least because of the unpredictable disease burden that differs markedly from year-to-year.^95^ As might therefore be anticipated, evaluations from different countries all show very large variability in the seasonal impact of QIVs. Analyses that project cumulative effects over multiple seasons based on antecedent virus circulation patterns are therefore most informative. The published dynamic models showed substantially greater improvement in health outcomes based on the use of QIVs as compared with the more conservative static models. However, although dynamic models better reflect the real-world impact of vaccination, dynamic transmission models are inherently more complex and require a greater degree of assumptions in terms of model inputs.

Influenza vaccines typically show reduced efficacy in the elderly due to immune senescence and novel TIVs developed for use in this age-group include an increased antigen dose or adjuvant to overcome this limitation. Modeling studies suggest that the benefits of enhanced TIV formulations in the elderly (high-dose or adjuvanted TIVs) may be as great, or greater than those provided by QIVs.^60,61,64^ This underscores the fact that in older adults, improvements in vaccine efficacy can be achieved by improving the immune response and by broadening coverage. However, even with high-dose TIV, efficacy against influenza B due to the lineage included in the vaccine is clearly higher than efficacy against influenza B due to the lineage absent from the vaccine.^96^ An enhanced QIV in this population appears to be the optimal vaccine choice.

Influenza vaccines have been in use since 1936 and the move from TIVs to QIVs is the most recent adaption of seasonal influenza vaccines in response to changes in global circulating influenza strains. Based on the available evidence from clinical trials, epidemiological studies and modeling, several countries have progressively issued recommendations preferentially recommending QIVs over TIVs. To address the co-circulation of B-lineage viruses or B-lineage mismatch, QIVs have been developed and are likely to lead to more stable vaccine effectiveness across seasons, providing broader protection than TIVs and contributing to influenza prevention worldwide. Availability of QIV efficacy data in children and estimates of vaccine effectiveness in coming seasons will provide complementary information on the potential added benefits of QIVs on influenza prevention, which may lead more countries to adopt definitive recommendations for QIVs use.

## Trademark statement

*FluLaval, Fluarix, Alpharix* and *Influsplit* are trademarks of the GSK group of companies. *Fluzone Quadrivalent* and *Fluzone Intradermal Quadrivalent* are trademarks of Sanofi Pasteur. *Flumist Quadrivalent* and *Fluenz Tetra* are trademarks of AstraZeneca.
